# Fasting conditions: Influence of water intake on clinical chemistry analytes

**DOI:** 10.11613/BM.2018.010702

**Published:** 2017-11-24

**Authors:** Silvia F. Benozzi, Gisela Unger, Amparo Campion, Graciela L. Pennacchiotti

**Affiliations:** 1 Bioquímica Clínica I, Departamento de Biología, Bioquímica y Farmacia, Universidad Nacional del Sur, Bahía Blanca, Argentina; 2 Hospital Municipal de Agudos ‘‘Dr. Leónidas Lucero’’, Bahía Blanca, Argentina

**Keywords:** fasting, water intake, blood sample collection, preanalytical variability, clinical chemistry tests

## Abstract

**Introduction:**

Currently available recommendations regarding fasting requirements before phlebotomy do not specify any maximum water intake volume permitted during the fasting period. The aim was to study the effects of 300 mL water intake 1 h before phlebotomy on specific analytes.

**Materials and methods:**

Blood was collected from 20 women (median age (min-max): 24 (22 - 50) years) in basal state (T_0_) and 1 h after 300 mL water intake (T_1_). Glucose, total proteins (TP), urea, creatinine, cystatin C, total bilirubin (BT), total cholesterol, high-density lipoprotein cholesterol, low-density lipoprotein cholesterol, triglycerides (Tg), uric acid (UA), high-sensitivity C-reactive protein, gamma-glutamyl transferase (GGT), aspartate-aminotransferase (AST), alanine-aminotransferase and lactate-dehydrogenase (LD) were studied. Results were analyzed using Wilcoxon test. Mean difference (%) was calculated for each analyte and was further compared with reference change value (RCV). Only mean differences (%) higher than RCV were considered clinically significant.

**Results:**

Significant differences (median T_0_ *vs* median T_1_, P) were observed for TP (73 *vs* 74 g/L, 0.001); urea (4.08 *vs* 4.16 mmol/L, 0.010); BT (12 *vs* 13 µmol/L, 0.021); total cholesterol (4.9 *vs* 4.9 mmol/L, 0.042); Tg (1.05 *vs* 1.06 mmol/L, 0.002); UA (260 *vs* 270 µmol/L, 0.006); GGT (12 *vs* 12 U/L, 0.046); AST (22 *vs* 24 U/L, 0.001); and LD (364 *vs* 386 U/L, 0.001). Although the differences observed were statistically significant, they were not indicative of clinically significant changes.

**Conclusions:**

A water intake of 300 mL 1 h prior to phlebotomy does not interfere with the analytes studied in the present work.

## Introduction

Reliability of laboratory results must be guaranteed through quality policies all along the biochemical process, including the pre-analytical phase, because of the fact that several medical decisions and procedures depend on them (*1*).

Although the pre-analytical phase is the most exposed to the risk of making mistakes in the laboratory, the importance of this phase is often underestimated and therefore its impact on results obtained is either unknown or minimized (*2*). Both the organization and management of the laboratory personnel involved in these procedures are therefore crucial because each stage of the biochemical process is subjected to variability (*3*). A critical issue that has been considered as an important source of error but is nonetheless essential in obtaining reliable results ensuring safety is patient preparation (*4*). Several studies have demonstrated that patients go to the laboratory for blood sample collection without adequate preparation. This is attributed to the fact that they lack information regarding the pre-analytical requirements for routine blood tests as well as to the absence of harmonization among laboratories (*5*-*8*). These two factors are indicative of the need for standardized, updated and evidence-based recommendations for patient preparation to minimize patient risks (*8*).

Previous research on the changes that biochemical and haematological markers undergo in response to postprandial state emphasizes the need for fasting prior to routine blood tests (*9*, *10*). However, fasting is considered either unnecessary or optional by some laboratories (*11*).

Within this context, the Working Group on Preanalytical Phase (WG-PRE) of the European Federation of Clinical Chemistry and Laboratory Medicine (EFLM) issued recommendations for patient preparation prior to blood sample collection for routine biochemical practices; namely to collect blood samples between 7 and 9 a.m. after a 12 h fasting period during which water intake is allowed, not to drink alcohol 24 hours before blood collection, and neither to smoke nor to drink caffeine-containing beverages during the morning of blood collection (*12*). Still, no specific recommendation on the maximum water volume intake allowed before blood collection has been issued to date, except that habitual water intake is permitted.

A recent consensus study has proposed that fasting is not necessary for the determination of the lipid profile (*13*). This recommendation was based on findings from several prospective studies revealing that whereas no changes were observed in high-density lipoprotein cholesterol (HDL) concentrations in response to a standard regular meal, plasma triglycerides (Tg) were found to slightly increase and total cholesterol and low-density lipoprotein cholesterol (LDL) values were observed to decrease although all these variations appeared to be clinically non-significant (*13*). The decrease in LDL values observed was attributed to the dilution effect derived from fluid-free intake. Still, a decrease in LDL in non-fasting individuals may lead to errors in the classification of cardiovascular risk and therefore in the indication of initiation of lipid-lowering therapy or in the evaluation of the changes that may occur during this therapy (*14*). In view of this, it is mandatory to study the effect and impact of water intake as a pre-analytical variable on routine biochemical laboratory tests.

Our hypothesis is that water intake during fasting may affect some laboratory parameters. The purpose of the present work is therefore to study the influence of a 300 mL water intake, which is equivalent to a glass of water, during fasting and 1 h before blood collection may have on some routine biochemical tests.

## Materials and methods

### Study design

This is an experimental longitudinal study carried out in a non-accredited laboratory (according to ISO 15189 regulations) at Hospital Municipal de Agudos “Dr. Leónidas Lucero” in Bahía Blanca city, province of Buenos Aires, Republic of Argentina, in October 2016. This study was conducted following the methodology previously published by different research groups (*9*, *10*, *15*).

### Subjects

The study population was composed of 20 female students of Biochemistry from the Universidad Nacional del Sur, Bahía Blanca, Argentina, who voluntarily agreed to participate in this study. Median age of volunteers was 24 (min-max: 22-50) years. All participants were informed of the aims of the present study. They stated that they had no health problems and that they were not taking any medication. They expressed their written consent. This study was approved by the Bioethics Committee of the above-mentioned hospital. Inclusion criteria were absence of chronic or acute pathologies. Exclusion criteria were medication treatment or physical activity 24 h before phlebotomy.

### Blood sampling

Blood samples for biochemical determinations in the basal state were collected on the same day, after nocturnal rest including a 12 h fasting period without water intake. The procedure applied was performed by a laboratory technician following the guidelines issued by the Clinical and Laboratory Standards Institute/National Committee for Clinical Laboratory Standards (CLSI/NCCLS) H03-A6 with adaptations suggested by Lima-Oliveira *et al*. (*16*, *17*). The first blood collection was performed at 9 a.m. following WG-PRE/EFLM recommendations, after the subjects had remained sitting for 15 minutes (*12*). Immediately after phlebotomy, volunteers drank a glass of water (300 mL) and remained sitting in the blood collection room for an extra hour, after which the second blood collection was carried out. Two blood samples (5 mL each) were thus obtained per individual: a basal one (T_0_) and another one 1 hour after water intake (T_1_).

Samples were collected via antecubital vein puncture using needles 21Gx1 (NEOJET, Zhejiang Ougian Medical Apparatus Co, Wenzhou, China) and syringes of 10 mL (Hongda, Jiangxi Hongda Medical Equipment Group Ltd., Nanchang, China). After collection, blood samples were distributed in 2.5 mL plastic tubes with lithium heparin additive (TECNON, Laboratorios Argentinos, Berisso, Argentina) and centrifuged at 1500xg for 15 minutes at room temperature.

Plasma was separated from packed red blood cells within the first 15 minutes after blood collection and immediately aliquoted in two polypropylene tubes with no additives (TECNON, Laboratorios Argentinos, Berisso, Argentina) for further analysis in different analysers. Blood samples were free from haemolysis or lipemia, as confirmed by the haemolysis and lipemia index evaluated in Vitros 4600.

Intake water was table water bottled by a local company (Aristu Hnos, Bahía Blanca, Argentina). It had been previously subjected to reverse osmosis, filtrated, ionized and ozonised to guarantee physical, chemical and biological quality for human consumption. Table 1 shows the chemical composition of the water used in our study provided by the manufacturer.

**Table 1:** Chemical composition of water provided by the manufacturer

**Analyte**	**Method**	**Concentration**
Sulphate	SM 4500 SO4-E (turbidimetric)	8 mg/L
Fluoride	SM 4500 FD (colorimetric SPADNS)	0.16 mg/L
Nitrate	SM 4500 NO3 E (cadmium reduction manual)	6.1 mg/L
Nitrite	SM 4500 NO2 B (spectrophotometric manual)	0.039 mg/L
Ammonia	SM 4500 NH3 C (titration)	< 0.1 mg/L
Chloride	SM 4500 Cl B (titrimetric silver nitrate)	0.840 mmol/L
Calcium	SM 3500 Ca D (titrimetric EDTA)	0.050 mmol/l
Sodium	Ion Selective Electrode	0.003 mmol/L
Magnesium	SM 3500 Mg E (colorimetric)	0.070 mmol/L
Iron	SM 3500 Fe D (colorimetric)	0.01 mg/L
Arsenic	SM 3500 As D (colorimetric)	< 0.005 mg/L
SM - standard methods for water & wastewater examination; available at http://www.standardmethods.org (*35*).		

### Biochemical tests

The analytes studied were: glucose (Glc), total proteins (TP), urea, creatinine (CREA), cystatin C, total bilirubin (BT), total cholesterol, HDL, LDL, Tg, uric acid (UA), high-sensitivity C-reactive protein (hsCRP), gamma-glutamyltransferase (GGT), aspartate-aminotransferase (AST), alanine-aminotransferase (ALT) and lactate-dehydrogenase (LD).

Biochemical determinations were performed by the Vitros® 4600 autoanalyser (Ortho Clinical Diagnostics, New Jersey, USA) with proprietary reagents and calibrators. Internal and external quality controls were included as part of the standard procedure. Measurements of the analytes cystatin C, hsCRP and LDL were performed using a Modular P800® analyser (Roche Diagnostics, Mannheim, Germany) following the same quality specifications. In both cases, determinations were made at the same time, which ensured the use of the same reagent lot, thus avoiding lot-to-lot variability. The analytical coefficient of variation (CV_A_) for each analyte was obtained using third party control material (BIORAD, Irvine, USA). Table 2 lists the analytes studied and the methods followed for their determination.

**Table 2:** Laboratory tests performed with corresponding methods

**Analyte (unit)**	**Method**
Glc (mmol/L)	Enzymatic/colorimetric-MicroSlide Technology
TP (g/L)	Colorimetric-MicroSlide Technology
Urea (mmol/L)	Enzymatic/colorimetric-MicroSlide Technology
CREA (µmol/L)	Enzymatic-MicroSlide Technology
Cystatin C (mg/L)	Turbidimetric immunoassay
BT (µmol/L)	Colorimetric-MicroSlide Technology
Cholesterol, total (mmol/L)	Enzymatic/colorimetric-MicroSlide Technology
HDL (mmol/L)	Direct assay-MicroSlide Technology
LDL (mmol/L)	Direct assay
Tg (mmol/L)	Enzymatic/colorimetric-MicroSlide Technology
UA (µmol/L)	Enzymatic/colorimetric-MicroSlide Technology
hsCRP (mg/L)	Turbidimetric immunoassay
GGT (U/L 37 °C)	Kinetic/UV-Microslide Technology
AST (U/L 37 °C)	Kinetic/colorimetric-MicroSlide Technology
ALT (U/L 37 °C)	Kinetic/UV-MicroSlide Technology
LD (U/L 37 °C)	Kinetic/UV-MicroSlide Technology
Glc - glucose. TP - total proteins. CREA - creatinine. BT - total bilirubin. HDL - high-density lipoprotein cholesterol. LDL - low-density lipoprotein cholesterol. Tg - triglycerides. UA - uric acid. hsCRP - high-sensitivity C-reactive protein. GGT - gamma-glutamyltransferase. AST - aspartate-aminotransferase. ALT - alanine-aminotransferase. LD – lactate-dehydrogenase.	

### Statistical analysis

As the number of individuals studied was lower than 30, data were processed using non-parametric statistics (*18*). Comparison of medians between T_0_ and T_1_ was made with the non-parametric Wilcoxon Signed Rank Test for paired samples. Statistical significance was set at P < 0.05. Statistical Package for Social Science for Windows (SPSS) software (Version 15.0. Chicago, IL, USA) was used for data analysis.

The clinically significant change was calculated for each analyte using the reference change value (RCV) equation as follows (*15*, *19*-*21*):
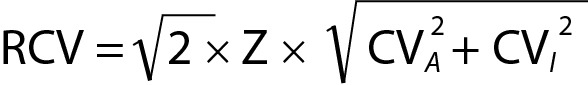
where Z is a constant for the level of statistical confidence (1.96 for α = 0.05), CV_I_ is the within-subject biological variation obtained from Westgard database, and CV_A_ is the analytical coefficient of variation from the laboratory internal quality control (*22*). All these data are included in Table 3.

**Table 3:** Variations in clinical chemistry analytes values after water intake

**Analyte**	**CV_A_** **(%)**	**CV_I_** **(%)**	**DSI** **(%)**	**Basal values (T_0_)**	**Values 1 h after** **water intake (T_1_)**	**P**	**Mean difference (%)**	**RCV (%)**
Glc (mmol/L)	1.4	4.5	2.3	4.8 (4.5-5.1)	4.9 (4.6-5.3)	0.104	1.5	13.0
TP (g/L)	0.9	2.8	1.4	73 (70-75)	74 (72-77)	0.001	2.2*	8.0
Urea (mmol/L)	3.0	12.1	6.1	4.08 (3.38-4.75)	4.16 (3.41-5.04)	0.010	2.9	34.6
CREA (µmol/L)	1.4	6.0	3.0	73.4 (68.9-77.8)	71.6 (66.3-77.8)	0.209	- 1.0	16.9
Cystatin C (mg/L)	0.6	5.5	2.5	0.75 (0.71-0.82)	0.75 (0.72-0.79)	0.375	- 1.1	15.3
BT (µmol/L)	5.8	21.8	10.9	12 (9-15)	13 (7-15)	0.021	5.2	62.5
Cholesterol, total (mmol/L)	1.8	6.0	3.0	4.9 (4.2-5.3)	4.9 (4.3-5.4)	0.042	1.2	17.2
HDL (mmol/L)	2.8	7.3	3.7	1.8 (1.3-1.9)	1.8 (1.4-2.0)	0.082	1.7	21.7
LDL (mmol/L)	1.6	7.8	3.9	2.6 (2.2-2.9)	2.5 (2.2-2.9)	0.129	- 1.3	22.1
Tg (mmol/L)	3.5	19.9	10.0	1.05 (0.79-1.62)	1.06 (0.75-1.43)	0.002	- 6.5	56.0
UA (µmol/L)	3.2	8.6	4.3	260 (220-290)	270 (220-290)	0.006	2.2	25.4
hsCRP (mg/L)	1.3	42.2	21.1	1.00 (0.42-2.27)	1.02 (0.42-2.13)	0.070	- 1.7	117.0
GGT (U/L 37 °C)	1.5	13.4	6.7	12 (10-17)	12 (10-18)	0.046	2.6	37.4
AST (U/L 37 °C)	5.5	12.3	6.2	22 (20-26)	24 (22-32)	0.001	9.2*	37.3
ALT (U/L 37 °C)	6.0	19.4	9.7	16 (13-22)	17 (12-22)	0.418	3.5	56.3
LD (U/L 37 °C)	3.6	8.6	4.3	364 (342- 394)	386 (364-418)	0.001	5.3*	25.8
Results are shown as median and interquartile range. P < 0.05 was considered statistically significant. *Mean difference (%) higher than DSI. CV_A_ - analytical coefficient of variation. CV_I_ - within-subject biological variation. DSI - desirable specification for imprecision derived from biological variation. T_0_ - basal; T_1_ - 1 h after 300 mL water intake. RCV - reference change value. Glc - glucose. TP - total proteins. CREA - creatinine. BT - bilirubin, total. HDL - high-density lipoprotein cholesterol. LDL - low-density lipoprotein cholesterol. Tg - triglycerides. UA - uric acid. hsCRP - high-sensitivity C-reactive protein. GGT - gamma-glutamyltransferase. AST - aspartate-aminotransferase. ALT - alanine-aminotransferase. LD – lactate-dehydrogenase.								

For each volunteer, the mean difference (%) of each analyte was calculated using the following formula (*23*-*27*):


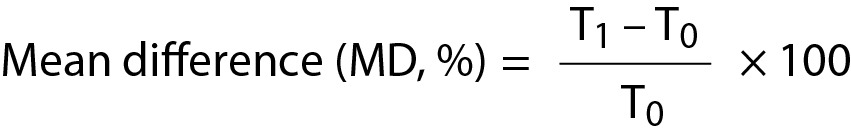


From these data, the mean value of the mean difference (MD, %) of each analyte was obtained and further compared with the corresponding RCV. A clinically significant difference was considered per analyte when MD (%) was higher than the respective RCV. For analytes with MD (%) higher than the desirable specification for imprecision (DSI) derived from biological variation, a graphical representation of the change or interference detected (interferogram) was created and expressed as MD (%) with respect to RCV (*22*, *28*).

## Results

Table 3 shows results from the present work, expressed as the median and interquartile range. After a 300 mL water intake 1 h before phlebotomy, significantly higher values (T_0_ median *vs* T_1_ median, P) were observed for urea (4.08 *vs* 4.16 mmol/L; 0.010), BT (12 *vs* 13 µmol/L; 0.021), total cholesterol (4.9 *vs* 4.9 mmol/L; 0.042), Tg (1.05 *vs* 1.06 mmol/L; 0.002), UA (260 *vs* 270 µmol/L; 0.006), and GGT (12 *vs* 12 U/L; 0.046).

**Figure 1:**
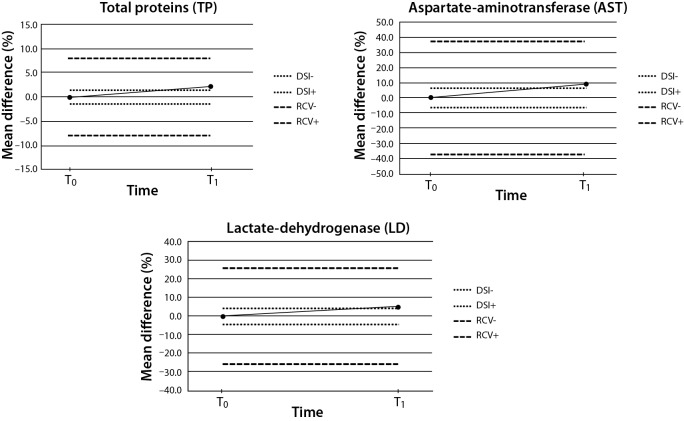
Water intake interferograms for total proteins, aspartate aminotransferase and lactate dehydrogenase. T_0_ - basal; T_1_ - 1 h after 300 mL water intake. DSI - desirable specification for imprecision derived from biological variation. RCV - reference change value.

As shown in Table 3 and Figure 1, after water intake significantly higher TP concentrations (73 *vs* 74 g/L; 0.001), AST (22 *vs* 24 U/L; 0.001) and LD activities (364 *vs* 386 U/L; 0.001) were observed. Still, unlike the previous cases, MDs (%) of these analytes exceeded their DSI (MD (%) vs DSI: 2.2 *vs* 1.4%; 9.2 *vs* 6.2%, and 5.3 *vs* 4.3%, respectively). Nonetheless, the comparison of MDs (%) with RCV showed that no clinically relevant changes occurred in the analytes studied.

## Discussion

Our results demonstrate that a 300 mL water intake 1 h before phlebotomy produces no clinically significant changes in the series of analytes assessed in our study.

Given the importance that reliable laboratory results have in medical decision-makings, instructions on fasting must be clearly and concisely given to patients to ensure sample quality. According to the general recommendations published in 2014 on the harmonization of water intake, water intake is permitted before phlebotomy, *i.e.* during fasting hours (*12*). However, little is known not only on how much water should be permitted but also on the effects of specific water intake volumes on laboratory parameters during fasting. In our study, an intake of a specific volume of water rather than a “habitual” or an “*ad libitum*” water intake was considered. The volume chosen was 300 mL which is equivalent to a regular glass of water that any patient may drink before phlebotomy.

Water absorption is a very rapid process. A recent study has shown that water appears in plasma and blood cells as soon as 5 min after water intake (*29*).

Torsdottir and Andersson demonstrated that a 300 mL water intake along with food after a 12 h fasting period increases blood Glc and insulin concentrations in healthy subjects, and only Glc in well-controlled diabetic patients (*30*). In our study, no significant changes were observed in Glc concentrations in the fasting state 1 h after intake of the same amount of water, thus suggesting that only concurrent food and water intake may influence Glc concentrations.

Furthermore, previous research conducted on individuals without previous fasting detected a decrease in LDL after food intake (*31*). The authors of this study attributed this decrease to a haemodilution effect caused by the liquids ingested. In contrast, our study showed that a 300 mL water intake 1 h prior to phlebotomy did not change LDL concentrations.

Several of the analytes studied in the present work were observed to undergo statistically significant changes. Special attention was paid to the differences observed in TP, AST and LD, which were higher than the DSI. In view of the need to evaluate the clinical relevance of such changes, both analytical and biological variability were taken into account. Our estimation of RCV, a reference point considered to be a more statistically structured approach than DSI for the determination of clinically relevant changes, revealed that none of the analytes studied underwent relevant changes (*15*, *32*).

Our findings clearly indicate the importance of objectively assessing the clinical significance of the statistically significant changes that may occur in an analyte concentration as a consequence of pre-analytical variability. That is, if differences are indeed recorded, to what extent are these differences relevant (*33*, *34*)? Decision makings based exclusively on the interpretation of the P value for the harmonization of patient preparation prior to phlebotomy may lead to erroneous conclusions.

A limitation of our study was the low number of participants as well as gender- and age-bias. Therefore, further research on a larger population will allow confirming the results derived from our small population-based study. In addition, taking into account that volunteers in the present study drank only 300 mL of water after a 12 h fasting period, it will be useful to investigate the influence of different water volume intakes during fasting on laboratory measurements to accurately determine the maximum water intake volume permitted during fasting.

The WG-PRE**/**EFLM states that although it is important to know the maximum volume of water intake permitted before phlebotomy, there is still no evidence recommending a specific water intake volume (*12*). In this sense, our finding showing that the ingestion of a regular glass of water 1 h before phlebotomy is accompanied by no clinically significant changes is therefore an important contribution to future research aiming at determining the water volume that could be permitted prior to some other clinical biochemical determinations.

Based on our results, it can be concluded that a 300 mL water intake 1 h prior to phlebotomy does not interfere in the analytes studied in the present work. This is the first study carried out on the effect of water intake during fasting hours on some biochemical determinations.
